# A biomechanical comparison study of a modern fibular nail and distal fibular locking plate in AO/OTA 44C2 ankle fractures

**DOI:** 10.1186/s13018-016-0435-5

**Published:** 2016-09-15

**Authors:** Paul J. Switaj, Daniel Fuchs, Mohammed Alshouli, Avinash G. Patwardhan, Leonard I. Voronov, Muturi Muriuki, Robert M. Havey, Anish R. Kadakia

**Affiliations:** 1 Department of Orthopaedic Surgery, Northwestern University, Chicago, IL USA; 2 Musculoskeletal Biomechanics Research Laboratory, Edward Hines Jr. VA Hospital, Chicago, IL USA

**Keywords:** Ankle fracture, Fibula, Intramedullary rod, Locked plate, Biomechanical

## Abstract

**Background:**

A lateral approach with open reduction and internal fixation with a plate is a very effective technique for the majority of distal fibular fractures. However, this open approach for ankle fixation may be complicated by wound dehiscence and infection, especially in high-risk patients. An alternative to plating is an intramedullary implant, which allows maintenance of length, alignment, and rotation and which allows for decreased soft tissue dissection. While there has been clinical data suggesting favorable short-term outcomes with these implants, there is no current biomechanical literature investigating this technology in this particular fracture pattern. This study sought to biomechanically compare an emerging technology with an established method of fixation for distal fibular fractures that traditionally require an extensive exposure.

**Methods:**

Ten matched cadaveric pairs from the proximal tibia to the foot were prepared to simulate an Arbeitsgemeinschaft für Osteosynthesefragen/Orthopaedic Trauma Association (AO/OTA) 44C2 ankle fracture and randomized to fixation with a distal fibular locking plate or intramedullary fibular rod. A constant 700-N axial load was applied, and all specimens underwent testing for external rotation stiffness, external rotation cyclic loading, and torque to failure. The syndesmotic diastasis, stiffness, torque to failure, angle at failure, and mode of failure were obtained from each specimen.

**Results:**

There was no significant difference in syndesmotic diastasis during cyclic loading or at maximal external rotation between the rod and plate groups. Post-cycle external rotation stiffness across the syndesmosis was significantly higher for the locking plate than the fibular rod. There was no significant difference between the rod and plate in torque at failure or external rotation angle. The majority of specimens had failure at the syndesmotic screw.

**Conclusions:**

In the present cadaveric study of an AO/OTA 44C2 ankle fracture, a modern fibular rod demonstrated less external rotation stiffness while maintaining the syndesmotic diastasis to within acceptable tolerances and having similar failure characteristics.

## Background

Ankle fractures comprise 9 % of all fractures [1, 2]. Surgical treatment of ankle fractures has changed little throughout the years and typically includes an extensile incision over the fibula with open reduction and internal fixation [3, 4]. However, wound infections affect up to 26 % of patients, and hardware complications affect up to 50 % of patients [5–7]. These complications occur more frequently in the elderly population, diabetics, and smokers [8–11]. Additionally, patients with fibular fractures associated with higher energy injuries, such as fractures of the distal tibia plafond, have high occurrences of wound complications [8, 9, 12].

An alternative to plating of fibular fractures is the use of an intramedullary implant. This technique allows re-establishment of length, alignment, and rotation of the distal segment while allowing a smaller incision with decreased soft tissue dissection. This is desirable in high-energy injuries with possible soft tissue compromise and older, diabetic, nicotine-using patients at high risk for wound complications. Additionally, patients with Arbeitsgemeinschaft für Osteosynthesefragen/Orthopaedic Trauma Association (AO/OTA) 44C-type fractures [13] (distal fibular fracture proximal to the distal tibiofibular syndesmosis) necessitate increased exposure of the fibula and may especially benefit from this minimally invasive technique. While intramedullary fixation has been used in the past, new technologies have expanded the potential applications of this technique and have shown favorable short-term outcomes with low rates of complications in traditional rotational ankle fractures [14–24]. This mode of fixation has also been proposed as an effective treatment in the setting of pilon fractures [25].

The majority of the limited clinical and biomechanical literature regarding this implant has focused on AO/OTA 44B-type fractures. An initial, unpublished biomechanical study showed improved fixation load to failure when comparing fibular rods to AO plating with a lag screw in AO/OTA 44B-type fibular fractures [26]. There has been no previous literature using a fibular rod in AO/OTA 44C-type fibular fractures (suprasyndesmotic), which are 27–44 % of operative ankle fractures, with AO/OTA 44C2 (multifragmentary) comprising 2.5–17 % of these fractures [4, 27, 28]. In these comminuted fractures, anatomic reduction and compression of fragments is not feasible. The goal in these fractures is to restore length and alignment of the fibula, the syndesmotic relationship, and the ankle mortise. Intramedullary fibular rods, such as the Acumed fibular rod (Acumed Fibula Rod System, Hillsboro, OR, USA), allow syndesmotic fixation, which is crucial in these injury patterns. In cases of proximal fractures and comminution where fixation of the fracture may be difficult and require extensive dissection for a plating construct, a fibular rod may offer superior outcomes by providing a stable construct with minimal soft tissue dissection.

The specific aim of this project is to evaluate the biomechanical properties of a fibular rod in comparison to bridge plating with a distal fibular locking plate (Acumed Low-profile Locking Lateral Fibula Plate, Hillsboro, OR, USA) in comminuted AO/OTA 44C-type fibular fractures. We hypothesize that a fibular rod will provide a biomechanically equivalent construct when compared to a lateral locking plate when evaluating external rotation stiffness, syndesmotic diastasis, and external rotation torque to failure. Evidence of biomechanical superiority or non-inferiority of the fibular rod in AO/OTA 44C fractures may lead to increased clinical investigation and more widespread use for this particular fracture pattern.

## Methods

### Specimen preparation and surgical technique

Twenty-four fresh-frozen cadaveric ankle specimens (12 matched pairs; 4 male and 8 female pairs; average age 50.1 years, range: 28–59 years) were obtained from the proximal tibia to the foot from the Biological Resource Center of Illinois (Rosemont, Illinois). Ten matched pairs were placed into two groups. One group received a traditional 13-hole distal fibular locking plate while the second group was instrumented with a locking fibular rod (3.6 mm × 180 mm). Prior to dissection and experimentation, the specimens were examined grossly and radiographically to exclude any specimens with prior ankle surgery or deformity. Given the variability in mechanical properties between cadaveric specimens, this study was designed to use matched pairs with statistical comparisons made between the left and right limbs to reduce the effect of specimen variation on statistical inferences.

For the first five matched pairs, the left limb received a fibular rod and the right limb received a 13-hole distal fibular locking plate. The fibular rod and locking plate were placed in the right and left limbs, respectively, of the last five matched pairs. Prior to testing, we removed the skin proximal to the ankle and exposed the fibula, interosseous membrane (IO), interosseous ligament (IL), anterior-inferior tibiofibular ligament (AITFL), posterior-inferior tibiofibular ligament (PITFL), and transverse ligament (TL).

Multiple biomechanical studies have been performed on AO/OTA 44C-type fractures in regard to syndesmotic stability with an intact fibula [29–34]. There is limited literature regarding the creation and biomechanical testing of an AO/OTA 44C-type fracture with a comminuted fibula [35]. Most biomechanical models of comminuted fractures are in more distal, AO/OTA 44B-type fractures [35–37]. Thus, in order to simulate the typical AO/OTA 44C2 fibular fracture, we marked the location of a fibular osteotomy at 7 and 8 cm proximal to the distal fibula tip and made transverse osteotomies at marked sites with an oscillating saw, removing an approximately 1-cm fibula cross section.

For the fibular rod group, we then placed the rod with the targeting device externally rotated 30° [38]. We drilled bi-cortically through two anterior-to-posterior interlocking holes and one tri-cortical hole for syndesmotic fixation parallel to the tibiotalar joint with the foot in neutral dorsiflexion.

**Fig. 1:**
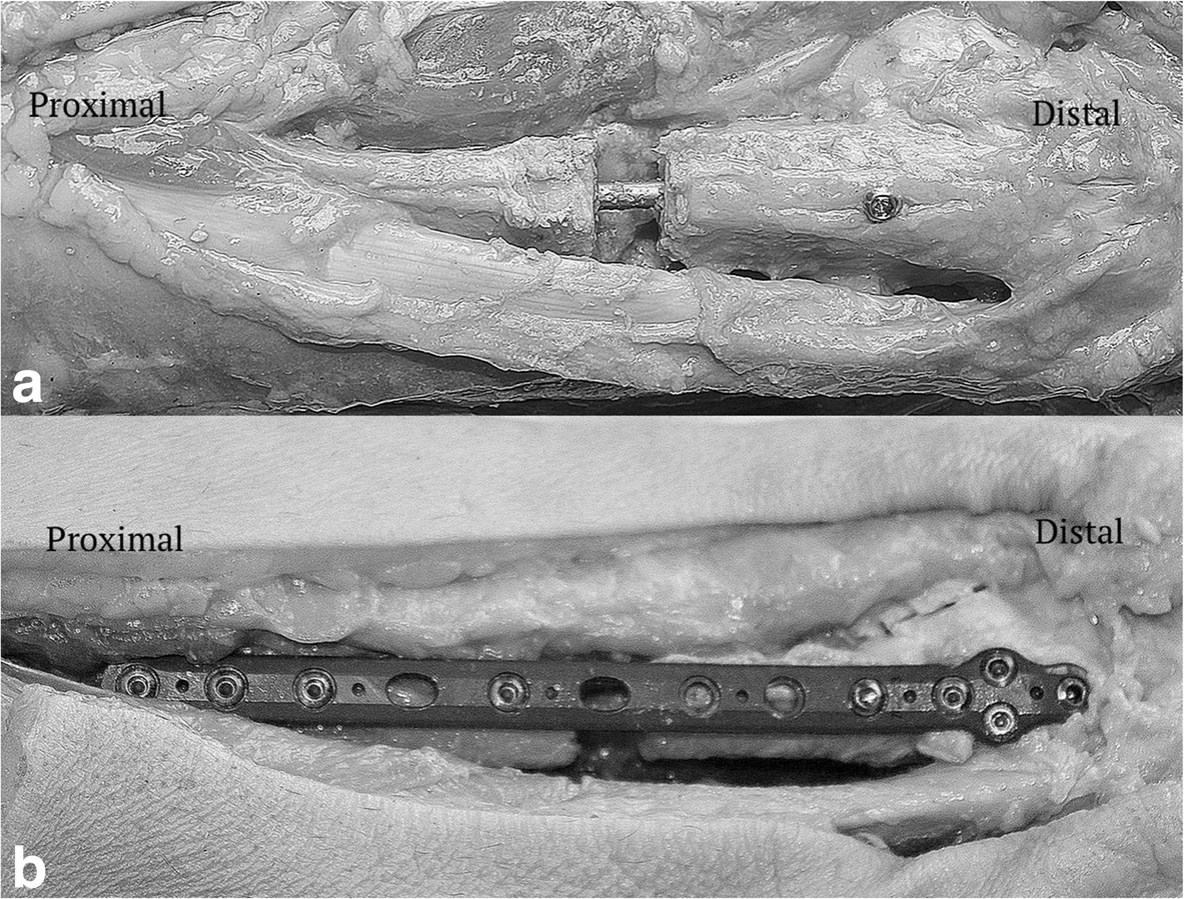
Post-fixation images of the fibular nail group (**a**) and the locked plate group (**b**)

**Fig. 2:**
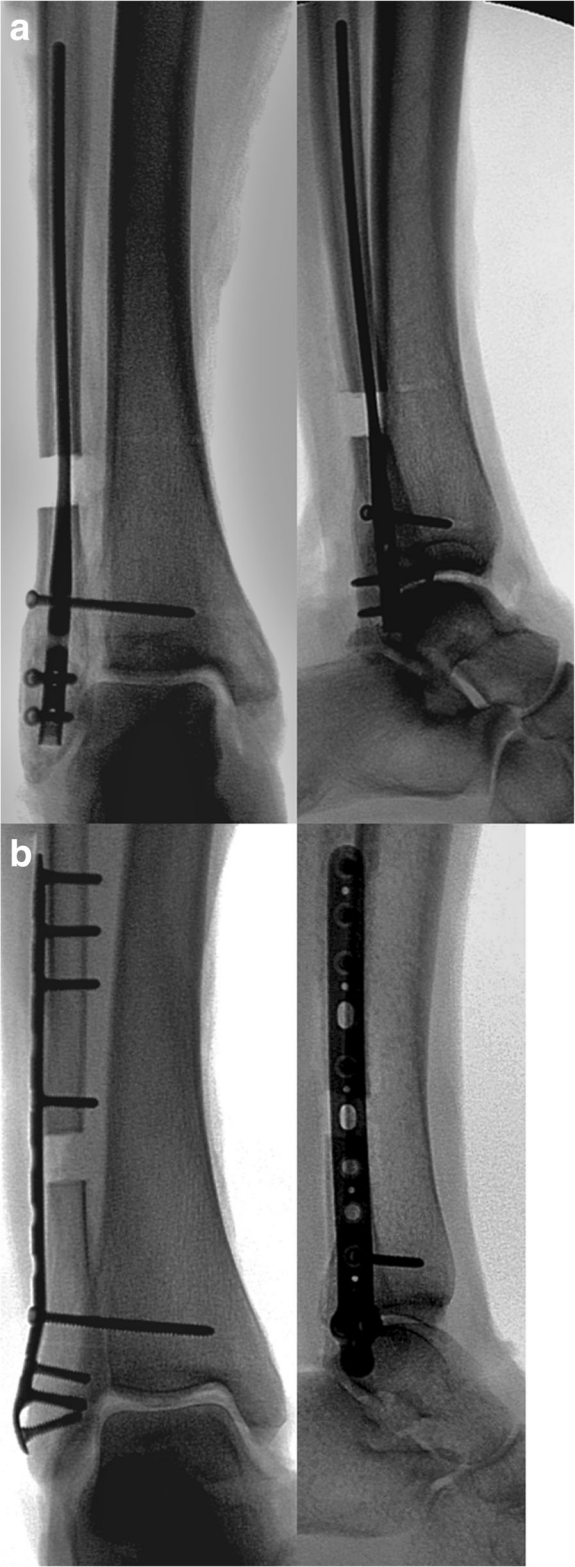
Post-fixation anteroposterior and lateral radiographs of the fibular nail group (**a**) and the locked plate group (**b**)

We inserted 3.5-mm non-locking cortical screws in the anterior-to-posterior interlocking holes. Once these screws were in place, we detached the interosseous membrane and interosseous ligament from fibula insertion using sharp dissection. We then inserted a 3.5-mm diameter syndesmotic screw in the previously drilled hole to gain tri-cortical purchase. After all the implants had been placed, we then detached the AITFL, PITFL, and TL from fibular insertion and lastly transected the deltoid ligament (Fig. 1a). A repeat radiograph was performed to demonstrate adequate fixation (Fig. 2a).

In the distal fibular locking plate group, we utilized a 13-hole low-profile lateral fibula locking plate. Prior to making the fibular osteotomy, the plate was appropriately positioned on the distal fibula. Using the locking drill guide, four unicortical holes were drilled in the distal cluster and four bicortical holes proximally. We drilled one tri-cortical hole for syndesmotic fixation parallel to the tibiotalar joint and externally rotated 30°. We then made our osteotomy as detailed above. The plate was re-positioned on the fibula, making sure that the predrilled holes were aligned such that the fibula was restored to the appropriate physiologic length. We inserted 3.5-mm locking screws into the predrilled holes both proximally and distally and a 3.5-mm diameter syndesmotic screw. Lastly, we detached the syndesmotic and deltoid ligaments and repeated the radiographs (Figs. 1b and 2b).

Lastly, two matched pairs (one male and one female) were used to quantify the biomechanics of the syndesmosis and intact fibula. For these four specimens, the deltoid ligament was transected but the syndesmotic complex of ligaments and fibula remained intact in order to determine the biomechanics in the native syndesmosis. This provided a standard for comparison for the rod and the plate fixation models.

### Biomechanical testing

**Fig. 3:**
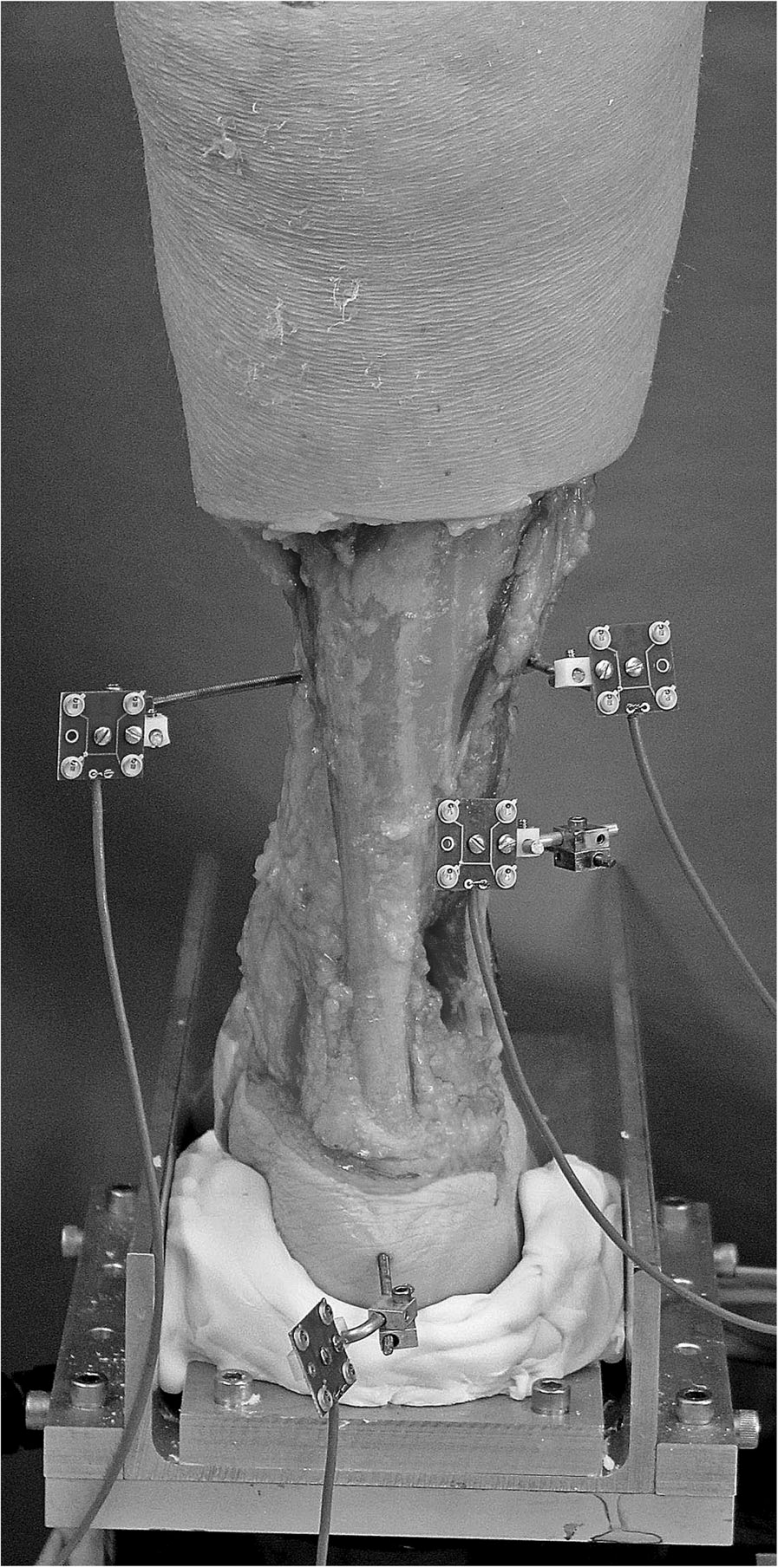
Biomechanical setup of a cadaveric specimen from posterior, demonstrating the custom plate and individual polymethylmethacrylate mold

The tibias of the specimens were fixed in custom cups using polymethylmethacrylate (PMMA) bone cement. A custom jig was used to ensure that the tibia was centered in the cup and that the mechanical loading axis of the tibia and the base of the cup were perpendicular. The cup was then attached to the actuator of a biaxial servohydraulic material testing machine (858 Mini Bionix, MTS Systems Corp., Eden Prairie, MN, USA). The feet were placed onto a custom testing apparatus, and an individual heel cup and midfoot support was molded using PMMA for each specimen to stabilize the foot on the plate (Fig. 3). Pins and screws were not used for fixation to the testing apparatus in order to eliminate the possibility of non-physiologic loading of individual bones.

Infrared light-emitting targets were rigidly fixed to the calcaneus, tibia, distal fibula, and proximal fibula. The three-dimensional position of these four targets was tracked during testing by an optoelectronic motion measurement system (Optotrak Certus, Northern Digital Inc., Waterloo, ON, Canada). Prior to testing, an optoelectronic three-dimensional digitizing probe was used to trace a series of points on the perimeter of the tibia at the level of the fibular osteotomy and on the distal fibula at the level of the syndesmotic screw. These points were digitized in relation to the optoelectronic target attached to the individual bones.

All the specimens underwent testing for pre-cycle external rotation stiffness, external rotation cyclic loading, post-cycle external rotation stiffness, and torque to failure. All testing was conducted with a 700-N constant compressive axial load applied to the leg.

The specimen was first tested to determine pre-cycle stiffness. After axial loading to 700 N, an external rotation torque was applied at 1°/s to a torque limit of 5 Nm. The specimen was cyclically loaded in external rotation while under the constant 700-N axial load 2000 times to a torque limit of 5 Nm under load control at a rate of 5 Nm/s. The post-cycle external rotation stiffness was then determined using the same protocol used for pre-cycle stiffness. Lastly, a torque to failure test was done for each specimen. An external rotation torque was applied to achieve rotation at a rate of 5°/s up to an actuator rotational displacement of 100° or an applied external rotation torque of 38 Nm. Axial load, external rotation torque, actuator rotation, actuator displacement, and the positions of the four infrared-emitting targets were recorded during the tests.

The pre- and post-cycle external rotation stiffness of the constructs was calculated from the applied external rotation torque and target rotation data. The stiffness was the slope of the line fit to the torque versus rotation data for an applied torque between 25 and 90 % of maximum (between 1.25 and 4.5 Nm). Fibular stiffness was calculated using the difference in rotation across the fracture site (between the targets distal and proximal to the fibular osteotomy). This stiffness (distal fibula to proximal fibula) gives an indication of how well the distal and proximal fibula are kinematically tied together by the construct and therefore, the amount of load transmitted across the osteotomy site. Syndesmosis stiffness was calculated using the difference in rotation across the syndesmosis (between the targets on the tibia and the distal fibula). This stiffness gives an indication of the quality of reduction of the syndesmosis.

Lastly, the maximum lateral translation of the distal fibula with respect to the tibia, termed the syndesmotic diastasis, was calculated for all the tests [31, 39, 40]. The positions of the probed points on the tibia and distal fibula during the tests were calculated using transformation matrices derived from the measured motion of the targets attached to the tibia and distal fibula [41]. At each time point, the lateral diastasis was calculated from the change in distance (relative to the beginning of the test) between the centroids of the tibia and fibular points. The maximum diastasis was determined from this data. The maximum external rotation torque or maximum external actuator rotation was obtained by reading the data at the point where the end of test was triggered.

### Statistical analysis

Statistical analyses were performed in specialized software (Systat, Cranes Software International Ltd, San Jose, CA). A repeated measures ANOVA was used to analyze differences between the two operative groups. On examination of the data, we found that three specimens had failed before any of the cyclical testing (one plate, two rod specimens). These were deemed to be outliers and were excluded from our final data evaluation. Thus, our total number of specimens was 17.

A post hoc power analysis was performed using 2-mm diastasis as a clinically relevant change. Utilizing eight pairs, the power was 42.5 % to detect a 2-mm gap. In order to achieve 80 % power to detect an effect size (difference in gap) of 2 mm, we would have needed to test 16 pairs of specimens.

## Results

### Syndesmotic diastasis and rotational stiffness without fixation

The maximum pre-cycle and post-cycle syndesmotic diastases were 1.4 ± 0.3 and 1.7 ± 0.4 mm, respectively. Thirty-eight Newton-meters of applied external rotation torque was achieved in all four intact state specimens during the external rotation torque to failure test.

Both measurements of external rotation stiffness increased from pre- to post-cycle. The distal fibula to proximal fibular stiffness increased from 1.2 ± 0.3 to 1.7 ± 0.2 Nm/degree, while distal fibula to tibia stiffness increased from 1.5 ± 0.5 to 2.0 ± 0.3 Nm/degree.

### Rotational stiffness after fixation

**Fig. 4:**
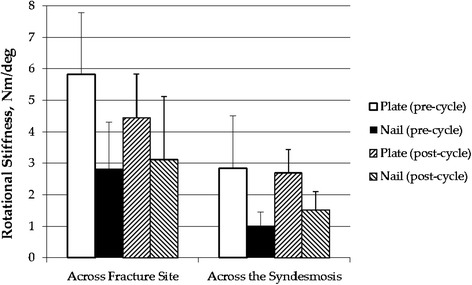
Bar graph depicting rotational stiffness data for both groups both pre-cycle and post-cycle

There was a significant difference between the external rotation stiffness pre-cycle for the locking plate versus the fibular rod. This was the case for the external rotation stiffness across the fracture site (5.8 versus 2.0 Nm/degree, *p* = 0.02) as well as external rotation stiffness across the syndesmosis (2.8 versus 1.0 Nm/degree, *p* = 0.048) (Table 1, Fig. 4).

**Table 1:** External rotation stiffness values, syndesmotic diastasis values, and failure properties for the fibular nail group and locked plate group

External rotation stiffness (Nm/degree)	Native	Fibular nail	Locked plate	*p v*alue
Pre-cyclic loading				
Across fracture	1.2 ± 0.3	2.0 ± 1.5	5.8 ± 2.0	0.02*
Across syndesmosis	1.5 ± 0.5	1.0 ± 0.4	2.8 ± 1.7	0.048*
Post-cyclic loading				
Across fracture	1.7 ± 0.2	3.1 ± 2.0	4.5 ± 1.4	0.23
Across syndesmosis	2.0 ± 0.3	1.5 ± 0.6	2.7 ± 0.8	0.03*
Syndesmotic diastasis (mm)				
Pre-cyclic loading	1.4 ± 0.3	1.4 ± 0.5	0.7 ± 0.2	0.08^a^
Post-cyclic loading	1.7 ± 0.4	2.4 ± 1.9	1.4 ± 0.5	0.08^a^
External rotation to failure	2.3 ± 0.8	7.6 ± 8.8	5.6 ± 3.0	0.465
Failure properties				
Torque to failure (Nm)		29.6 ± 4.8	28.1 ± 6.2	0.46
Angle to failure (degree)		91.6 ± 15.8	93.5 ± 18.2	0.73
Mode to failure (# of specimens)				
	Screw loosening	4	7	
	Soft tissue damage	1	1	
	Fibular fracture	3	1	

*Statistically significant

^a^The effect of construct on gap considers the pre- and post-cycle data together using repeated ANOVA

The external rotation stiffness post-cycle was significantly higher for the locking plate than the fibular rod in regard to stiffness across the syndesmosis (2.7 versus 1.5 Nm/degree, *p* = 0.03), but not across the fracture site (4.5 versus 3.1 Nm/degree, *p* = 0.23).

### Syndesmotic diastasis after fixation

**Fig. 5:**
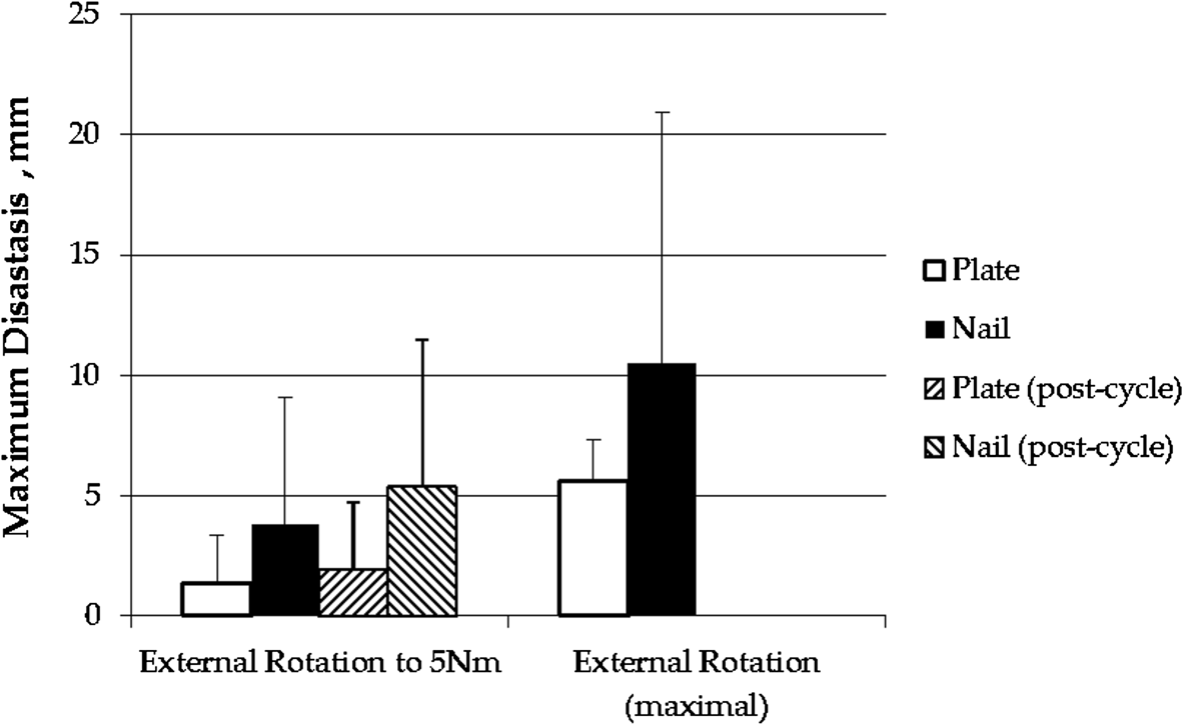
Bar graph depicting syndesmotic diastasis data for both groups both pre-cycle and post-cycle

With 5 Nm of applied external rotation torque, there was no significant difference in syndesmotic diastasis between the rod and plate groups both pre-cycle (1.4 versus 0.7 mm) and post-cycle (2.4 versus 1.4 mm) using repeated measures ANOVA (*p =* 0.08). During external rotation testing to failure, there was no significant difference in the diastasis (7.6 mm for the rod versus 5.6 mm for the plate, *p* = 0.465) (Table 1, Fig. 5).

### Failure properties after fixation

The primary trigger for the end of test in the external rotation to failure tests was reaching an actuator external rotation of 100° (Table 1). This occurred in 10 of the 17 specimens (five fibular rods and five locking plates). Five of the remaining tests (5 of 17) ended when the external torque limit of 38 Nm was reached (two fibular rods and three locking plates). The remaining tests (2 of 17) ended below 38 Nm and below 100° (one fibular rod and one locking plate). These two specimens were also found to have a fibular fracture. All the intact specimens (4 of 4) reached the external rotation torque limit of 38 Nm.

There was no significant difference between the rod and plate in torque at failure (29.6 ± 4.8 versus 28.1 ± 6.2 Nm, *p* = 0.46) or external rotation angle (91.6° ± 15.8° versus 93.5° ± 18.2°, *p* = 0.73).

An examination of the specimens in the operative group after testing found that the syndesmotic screw had loosened in 11 of the 17 specimens (seven locking plates and four fibular rods). Fibular fracture occurred in 4 of the 17 specimens (one locking plate and three fibular rods). There was soft tissue damage in 2 of the 17 specimens (one locking plate and one fibular rod).

## Discussion

Ankle fractures are a common orthopedic injury that occur at a rate of 187 per 100,000 person-years. They are the fourth most common fracture to require operative repair, which is recommended for unstable ankle fractures [42–46]. Wound complications can occur more frequently in certain patient populations and have a negative effect on long-term functional outcomes [5–7, 10–12, 47].

The use of intramedullary implants, such as screws, smooth rods, and the Kirschner wires, allows for decreased soft tissue dissection and has been a potential alternative to plating of fibular fractures [15, 20–22, 25]. Recent advancements in these intramedullary devices that allow for interlocking screw fixation have garnered an increased interest in expanding their use. To date, however, there is limited support in the literature [14–18, 48]. In the largest series to date, Bugler et al. reported on 105 patients treated for an unstable ankle fracture with the same fibular rod utilized in this study and showed good radiographic and functional outcomes at 6-year follow-up [16]. Recently, a prospective trial compared a fibular rod to plate fixation in non-comminuted fractures without syndesmotic injury and found significantly fewer complications and better functional scores at 1-year follow-up in the rod group [17].

Despite the increased interest in intramedullary fibular fixation, there have been no published biomechanical studies investigating the biomechanical properties of the modern fibular rod at present. While most of the existing literature examines the use of the fibular rod in Weber B fibular fractures, we feel that a potentially beneficial fracture pattern to utilize this fixation would be in AO/OTA 44C2 ankle fractures, which typically requires a large dissection and syndesmotic fixation [49, 50].

Several investigations suggest that increased syndesmosis width can lead to poor outcomes [51, 52]. Leeds and Ehrlich reported a significant increase in arthrosis of the ankle joint if the syndesmotic diastasis was greater than 2 mm compared to the normal contralateral side after undergoing open reduction and internal fixation [53]. Additional studies have demonstrated that the inability to obtain, and maintain, a proper syndesmotic reduction can lead to poor outcomes [54, 55].

It is important to define the normal functional anatomy of the distal tibiofibular joint when examining biomechanical literature. Beumer et al. showed that application of a 75-Nm external rotation moment on the foot of 11 healthy volunteers caused coronal plane translation between 0 and 2.5 mm and stated that these data can be used as normal reference values for studies of patients with suspected syndesmotic injuries [56]. Other studies have demonstrated similar physiologic motion at the distal tibiofibular joint [29]. Our study was consistent with this data, with our native specimens showing an average of 1.7 mm of diastasis post-cyclic loading.

Unfortunately, since there is no published biomechanical data on the modern fibular rod, we are unable to directly compare our results with those of other investigators. However, when inspecting the previous biomechanical literature in fracture models, Nousiainen et al. used a Weber C model and a similar biomechanical setup as the current study and showed that the change in syndesmosis width was between 0.5 and 1.25 mm in pre-cyclic loading when using rigid screw fixation of either three or four cortices, but did not utilize post-cyclic testing [32]. When examining dynamic fixation methods in a cadaveric model under cyclic loads, Ebramzadeh et al. showed that diastasis remained less than 2 mm in all specimens [57]. Stein et al. demonstrated that, under axial loads, syndesmotic diastasis can increase to 2.42 mm, even with screw fixation [58].

In the present study, when excluding outliers, both the fibular rod and locked plate demonstrated values in this range under pre-cyclic loading (1.4 ± 0.5 and 0.7 ± 0.2, respectively). The post-cyclic loading data shows that the fibular rod had a mean of 2.4 ± 1.9 mm of diastasis, which is only 0.7 mm greater than the native syndesmosis in the study and still within normal variation shown by Beumer et al. [56]. The locked plate group demonstrated less diastasis under cyclic loads than even the native group, suggesting the construct limited the normal physiologic diastasis to some degree. Thus, when analyzing our data in the context of this historic cadaveric data, we suggest that both fixation constructs provide adequate stability to limit syndesmotic diastasis during the immediate postoperative period. However, the increased diastasis with the nail would suggest that any change in this value would place it outside the normal variation. Thus, it is imperative that patients adhere to a strict non-weight bearing protocol while the ligamentous healing occurs to prevent any significant changes in the syndesmotic position.

In regard to external rotation stiffness, in our study, the fibular rod was found to have less external rotation stiffness than the locking plate. This was true when measuring stiffness across the fracture site and across the syndesmosis. It is not surprising that the plate group demonstrated this increased external rotation stiffness, since the distal locking plate has multiple points of fixation both proximal and distal to the fracture site. The fibular rod has no proximal interlocking fixation to provide enhanced rotational control at the fracture site, but does provide stability to the distal segment through the syndesmotic interlocking screw. In this fracture pattern where the fracture occurs above the level of the syndesmosis, the main focus would be on the rotational control of the distal segment which includes the tibiofibular articulation. The clinical significance of this increased external rotation stiffness is unclear but again would suggest the importance of proper postoperative weightbearing precautions in the nail group

In the present study, there was no significant difference in the failure properties between the two groups in either torque to failure or angle at failure. The values obtained for both groups were consistent with prior data examining syndesmotic fixation with both dynamic fixation and screw fixation by Ebramzadeh et al. and provided failure torques well above 20 Nm, exceeding likely torques applied in casts during healing [57]. Loosening of the syndesmotic screw was the primary observation in both operative groups. Distal fibular fracture inferior to the syndesmotic screw occurred more often in the fibular rod group. We hypothesize that this may be due to the lack of rotational control in the proximal segment, which could impart increased stress onto the distal interlocking screws.

There are several important limitations to note in the current study. Importantly, we excluded three specimens from our data analysis because their values fell far outside the mean values of the remaining specimens and demonstrated early catastrophic failure under pre-cyclic loads. This could be indicative of the potential surgeon-related difficulties with new technologies. However, statistical analysis was performed both including and excluding the outliers, and the comparative results were not changed substantially. Our post hoc power analysis revealed that we would have needed 32 specimens in order to achieve 80 % power to detect a 2-mm difference. Given financial constraints, this unfortunately was not attainable. Additionally, we did not examine the biomechanical properties of a non-locking plate and screw construct in this study, which is an acceptable form of fixation in this fracture pattern.

## Conclusions

In the present cadaveric study of an AO/OTA 44C2 ankle fracture, a modern fibular rod demonstrated inferior external rotation stiffness while maintaining the syndesmotic diastasis to within acceptable tolerances and having similar failure characteristics. We believe that the fibular rod can be utilized in select patient populations given that proper postoperative restrictions are placed.

## Abbreviations

AO/OTA: Arbeitsgemeinschaft für Osteosynthesefragen/Orthopaedic Trauma Association
N: Newton
Nm: Newton-meter
Mm: Millimeter
IO: Interosseous membrane
IL: Interosseous ligament
AITFL: Anterior-inferior tibiofibular ligament
PITFL: Posterior-inferior tibiofibular ligament
TL: Transverse ligament
PMMA: Polymethylmethacrylate
